# Age at Menarche and Natural Menopause and Number of Reproductive Years in Association with Mortality: Results from a Median Follow-Up of 11.2 Years among 31,955 Naturally Menopausal Chinese Women

**DOI:** 10.1371/journal.pone.0103673

**Published:** 2014-08-04

**Authors:** Xiaoyan Wu, Hui Cai, Asha Kallianpur, Yu-Tang Gao, Gong Yang, Wong-Ho Chow, Hong-Lan Li, Wei Zheng, Xiao-Ou Shu

**Affiliations:** 1 Division of Epidemiology, Department of Medicine, Vanderbilt Epidemiology Center, and Vanderbilt-Ingram Cancer Center, Vanderbilt University School of Medicine, Nashville, Tennessee, United States of America; 2 Department of Epidemiology, Shanghai Cancer Institute, Shanghai, China; 3 Occupational Epidemiology Branch, National Cancer Institute, National Institutes of Health, Bethesda, Maryland, United States of America; Geisel School of Medicine at Dartmouth College, United States of America

## Abstract

**Background:**

Studies conducted in Western countries suggest that early age at menarche and early age at menopause are both associated with increased total mortality, but limited data are available for Asian populations. We examined associations of age at menarche and natural menopause and duration of the reproductive span with mortality in a population-based cohort study of Chinese women.

**Methods:**

We evaluated the effects of age at menarche, age at natural menopause, and number of reproductive years on total and cause-specific mortality among 31,955 naturally menopausal Chinese women who participated in the Shanghai Women's Health Study, a population-based, prospective cohort study.

**Results:**

A total of 3,158 deaths occurred during a median follow-up of 11.2 years. Results from Cox proportional hazards models showed that younger age at menopause (<46.64 years) was associated with higher risk of total mortality (*P_trend_*  = 0.02). Younger age at menarche (<14 years) was associated with higher risk of mortality from stroke (*P_trend_*  = 0.03) and diabetes (*P_trend_* = 0.02) but lower risk of mortality from respiratory system cancer (*P_trend_*  = 0.01). Women with a shorter reproductive span had lower risk of mortality from gynecological cancers (*P_trend_* = 0.03).

**Conclusions:**

Our study found that menstrual characteristics are important predictors of mortality, suggesting an important role of sex hormones in biological aging.

## Introduction

The interval between menarche and menopause defines a woman's natural reproductive span [1]. Due to exposure to different hormonal environments, early or late onset of these events may be associated with an increased risk of many chronic health problems. Early menopause has been associated with increased risk of mortality from all causes [2]–[4], cardiovascular disease (CVD), and coronary heart disease (CHD) [4]–[7], but has not been associated with mortality from stroke or ischemic heart disease (IHD) [4], [8]. Early menarche has been associated with increased risk of mortality from all causes and CVD [9]–[11]. Other than CVD, few studies have examined the association of age at menarche and menopause with cause-specific mortality, and the results have been inconsistent [3], [4].

These findings are primarily based on studies conducted in Western populations. The few studies conducted among Asian women [12]–[15] were limited by one or more of the following factors: small sample size, focused only on cardiovascular cause-specific mortality, included only one component of reproductive information (e.g., age at menarche or age at menopause), no adjustment for other menstrual variables, or inadequate information on the cause of menopause. For example, Cui *et al.* found no statistically significant association of age at menarche, age at menopause, or number of reproductive years with mortality from CVD in a prospective study of 37,965 Japanese women [16]. Among 2,685 Korean women, Hong *et al.* observed that early menopause was associated with increased risk of mortality from all causes, CVD, and cancer [17]. Asian women, including Chinese women, have different menstrual and reproductive patterns as well as different lifestyle factors compared with women living in the United States and other Western countries. It is unclear whether menstrual characteristics are associated with mortality among Asian women.

The objective of the present study was to examine the effects of age at menarche, age at natural menopause, and the duration of the reproductive span on total and cause-specific mortality among women who participated in the Shanghai Women's Health Study, a large, population-based cohort study conducted in China.

## Methods

### Ethics Statement

All participants provided written informed consent, and the study protocols were approved by the Institutional Review Boards of Vanderbilt University and the Shanghai Cancer Institute.

### Study population

The Shanghai Women's Health Study is an ongoing, population-based, prospective cohort study, which enrolled 74,941women aged 40–70 years in 7 urban districts of Shanghai between 1997 and 2000 (participation rate: 92%). An in-person interview was conducted at baseline at an in-home visit, using a structured questionnaire to collect information on demographics, dietary habits, physical activity, occupational history, personal habits, history of chronic disease and surgery, family cancer history, reproductive history, and hormone use. Anthropometric measurements, including height, weight, and waist and hip circumferences, were also taken at baseline by trained personnel using standardized protocols. Details of the baseline survey have been published elsewhere [18].

Three biennial in-person follow-up surveys were conducted for all living cohort members at in-home visits conducted between 2000 to 2002 (response rate: 99.8%), 2002 to 2004 (response rate: 98.7%), and 2004 to 2007 (response rate: 96.7%) to collect information on occurrence and mortality from cancer, CVD, and other chronic diseases. The outcome information was supplemented by annual linkage to the population-based Shanghai cancer and vital statistics registries, which routinely collect data on vital status and cancer diagnosis. The out migration rate in Shanghai is extremely low, and thus, our follow-up for survival status for the study is virtually 100%.

At the time of enrollment, 37,168 participants (49.6%) were postmenopausal. We excluded from the analysis all women with missing data on menarche (*N* = 3); body mass index (BMI) or waist-to-hip ratio (WHR) (*N* = 16); women who were lost to follow-up shortly after study enrollment (*N* = 6); were menopausal due to hysterectomy, ovariectomy, or other causes (*N* = 4,114); or had ever used hormone replacement therapy (HRT) (*N* = 1,074). Thus, 31,955 women (42.6% of all participants and 85.9% of postmenopausal women) who experienced natural menopause were included in the current study.

### Menstrual variables

At baseline, each participant was asked her age at the time of her first period, which was recorded as age at menarche. Menopausal status was defined based on the World Health Organization's definition of menopause as the absence of menstruation for ≥12 months. The age at which menopause occurred and the reasons for its occurrence (natural menopause, hysterectomy or ovariectomy, or another cause) were recorded at baseline. The number of reproductive years (i.e., the “reproductive span”) was then calculated as the interval between age at menarche and age at menopause.

### Outcome definition

The outcome for this study was death from any cause that occurred after the baseline survey but before December 31, 2009. For surviving participants, follow up time was censored at December 31, 2009. The International Classification of Diseases, 9th Revision (ICD-9) [19], was used to define the cause of death, which was classified into CVD (ICD codes:390–459), IHD (ICD codes: 410–414), stroke (ICD codes: 430–438), diabetes (ICD code: 250), any cancer (ICD codes: 140–208), gynecological cancers (ICD codes: 174, 179–183), digestive system cancers (ICD codes: 150–159), and respiratory system cancers (ICD codes:160–165).

### Measurement of selected potential confounders

Socio-demographic information collected at baseline using the structured questionnaire included age at study enrollment (years), level of education (none, elementary school, middle school, high school, college or above), occupation (professional, clerical, manual laborer, housewife/retired), family income in *yuan*/year (<10,000; 10,000–19,999; 20,000–29,999;>30,000), marital status (yes: married; no: single, widow, divorced, separated), current smoking (yes, no), current drinking (yes, no), age at menarche (years), age at menopause (years), and number of live births. BMI (kg/m^2^) was calculated from weight (kg) and height squared (m^2^). WHR was calculated from waist circumference (cm) and hip circumference (cm). Physical activity was measured in metabolic equivalents (MET-h/day/year) based on a validated physical activity questionnaire [20].

### Statistical analyses

Person-years of follow-up for each participant were calculated from the date of the baseline interview through the date of death or December 31, 2009. Women were divided into quintiles of menstrual variables based on their distributions in the study population. The following menstrual variable categories were used as the reference groups in the analyses: aged 15 years for age at menarche, aged 48.80–50.15 years for age at natural menopause, and 30.16–32.45 years for number of reproductive years. These reference groups were chosen because they cover the medians of each menstrual variable (i.e., age at menarche, 15 years; age at natural menopause, 49.58 years; and number of reproductive years, 31.33 years).

Selected demographic and other factors were compared across categories of age at menopause, using analysis of variance (ANOVA) for continuous variables and chi-square tests for categorical variables. Cox proportional hazards models stratified by birth calendar year were employed to estimate hazard ratios (HR) and their 95% confidence intervals (95% CIs) for each group of menstrual variables adjusting for age at study enrollment (years), education (4 categories), occupation (4 categories), income (4 categories), marital status (yes/no), BMI (kg/m^2^), WHR (continuous), regular exercise (met/hour/year), current smoking (yes/no), current alcohol consumption (yes/no), number of live births, age at menarche (years, included in the Cox models for age at menopause and reproductive years), and age at menopause (years, included in the Cox models for age at menarche). Linear trends were tested across categories of menstrual variables by modeling the median values in each category.

SAS (version 9.2, SAS Institute, Inc., Cary, NC) was used in all analyses and two-sided *P*-values <0.05 were considered statistically significant.

## Results

Women in the lowest quintile for age at menopause were more likely to be current smokers, current alcohol drinkers, and not married and to have lower household income; they were less likely to have a professional job; and they had fewer live births compared with other groups. Women in the highest quintile for age at menopause were older, were more likely to exercise regularly, were younger at menarche, had higher BMI, had more reproductive years, and were less likely to have attained a high school education compared with other groups. Differences in WHR across quintiles of age at menopause approached a statistically significant level (Table 1).

**Table 1:** Population characteristics by age at menopause (years), Shanghai Women's Health Study.

Characteristics	Quintiles of age at menopause (years)					All	*P* value^a^
	<46.64	46.64–48.79	48.80–50.15	50.16–52.03	≥52.04	(*N* = 31,955)	
	(*N* = 6,389)	(*N* = 6,375)	(*N* = 6,363)	(*N* = 6,425)	(*N* = 6,403)		
Age at recruitment (years), mean (SD)	59.65(7.43)	60.49(6.46)	60.87(5.94)	61.00(5.46)	62.03(4.54)	59.65(7.43)	<0.001
Body mass index, mean (SD)	24.49(3.77)	24.57(3.57)	24.63(3.66)	24.72(3.53)	25.01(3.62)	24.49(3.77)	<0.001
Waist-to-hip ratio, mean (SD)	0.826(0.057)	0.824(0.056)	0.825(0.056)	0.825(0.055)	0.827(0.054)	0.825(0.056)	0.07
Education, high school (%)	16.67	18.95	19.35	21.94	14.92	20.07	<0.001
Occupation, professional (%)	16.95	25.31	25.14	33.36	28.27	28.30	<0.001
Family income, < 10,000 *yuan*/year (%)	23.08	21.23	20.82	19.80	22.77	20.60	<0.001
Regular exercise (%)	46.58	45.80	48.28	51.24	52.11	49.34	<0.001
Current smoking (%)	7.12	4.96	3.88	2.84	3.14	3.61	<0.001
Current alcohol consumption (%)	3.70	1.93	2.09	1.95	3.57	2.12	<0.001
Married (%)	79.34	83.62	82.86	83.77	80.87	83.12	0.002
Age at menarche (years), mean (SD)	15.21(1.88)	15.27(1.79)	15.27(1.82)	15.17(1.82)	15.16(1.79)	15.21(1.88)	0.02
Number of reproductive years, mean (SD)	25.53(4.62)	29.18(4.24)	30.74(4.46)	32.16(4.71)	35.18(4.93)	25.53(4.62)	<0.001
Number of live births, mean (SD)	2.48(1.46)	2.60(1.45)	2.58(1.38)	2.57(1.31)	2.69(1.28)	2.48(1.46)	<0.001

^*a*^ANOVA for continuous variables and Chi-square test for categorical variables.

During a median follow-up of 11.2 years, 3,158 women died. These deaths included 1,001 from all CVD (IHD: 236, stroke: 553), 246 from diabetes, 1,321 from all cancers (gynecological cancer: 174, digestive system cancer: 655, respiratory system cancer: 271), and 590 from other causes.

Table 2 presents age-and multivariable-adjusted HRs of total and cause-specific mortality according to age at menopause. After adjustment for potential confounding factors, younger age at menopause (<46.64 years) was associated with higher risk of total mortality (HR (95%CIs):1.16 (1.04, 1.29), *P*_trend_ = 0.02). Compared with the reference group (aged 48.80–50.15 years at menopause), being younger (<46.64 years) or older (50.16–52.03 years) at menopause was associated with higher risk of mortality from all cancers (HR (95%CIs): 1.27 (1.06, 1.51) for women aged <46.64 years and 1.34 (1.13,1.60) for women aged 50.16–52.03 years). Additionally, being younger (<46.64 years) or older (50.16–52.03 years) at menopause than the reference group was also associated with higher risk of mortality from digestive system cancers (HR (95%CIs): 1.43 (1.11, 1.84) for women aged <46.64 years and 1.38 (1.07, 1.77) for women aged 50.16–52.03 years). All of these associations were statistically significant. However, the trend for increasing mortality with increasing or decreasing age at menopause was not significant for all cancers combined or for digestive system cancers.

**Table 2:** HRs (95%CIs) of age at menopause (years) with total and cause-specific mortality, Shanghai Women's Health Study.

Cause of death		Quintiles of age at menopause (years)					*P_trend_* c
		<46.64	46.64–48.79	48.80–50.15^a^	50.16–52.03	≥52.04	
		(*N* = 6,389)	(*N* = 6,375)	(*N* = 6,363)	(*N* = 6,425)	(*N* = 6,403)	
All causes							
	Number of deaths	675	629	606	652	596	
	Age-adjusted hazard ratio	1.17(1.05,1.31)	1.04(0.93,1.17)	1.00	1.08(0.97,1.21)	0.93(0.83,1.04)	<0.001
	Multivariate hazard ratio^b^	1.16(1.04,1.29)	1.03(0.92,1.15)	1.00	1.11(0.99,1.24)	0.99(0.88,1.11)	0.02
All CVD							
	Number of deaths	215	201	219	172	194	
	Age-adjusted hazard ratio	1.03(0.85,1.24)	0.92(0.76,1.11)	1.00	0.80(0.65,0.98)	0.84(0.69,1.02)	0.02
	Multivariate hazard ratio^b^	1.01(0.83,1.22)	0.90(0.74,1.09)	1.00	0.82(0.67,1.00)	0.89(0.73,1.08)	0.15
Ischemic heart disease							
	Number of deaths	52	47	43	42	52	
	Age-adjusted hazard ratio	1.27(0.85,1.90)	1.10(0.72,1.66)	1.00	1.00(0.65,1.52)	1.15(0.76,1.72)	0.50
	Multivariate hazard ratio^b^	1.24(0.83,1.86)	1.07(0.71,1.62)	1.00	1.02(0.66,1.56)	1.23(0.82,1.84)	0.84
Stroke							
	Number of deaths	110	104	133	98	108	
	Age-adjusted hazard ratio	0.88(0.68,1.13)	0.79(0.61,1.02)	1.00	0.75(0.58,0.97)	0.77(0.60,1.00)	0.37
	Multivariate hazard ratio^b^	0.86(0.66,1.11)	0.77(0.60,1.00)	1.00	0.76(0.59,0.99)	0.81(0.63,1.04)	0.71
Diabetes							
	Number of deaths	53	54	51	43	45	
	Age-adjusted hazard ratio	1.08(0.74,1.59)	1.06(0.72,1.55)	1.00	0.86(0.57,1.29)	0.84(0.56,1.25)	0.13
	Multivariate hazard ratio^b^	1.06(0.72,1.56)	1.02(0.70,1.50)	1.00	0.90(0.60,1.36)	0.95(0.63,1.42)	0.47
All cancers							
	Number of deaths	273	247	227	300	274	
	Age-adjusted hazard ratio	1.27(1.07,1.52)	1.10(0.92,1.32)	1.00	1.31(1.11,1.56)	1.14(0.95,1.36)	0.52
	Multivariate hazard ratio^b^	1.27(1.06,1.51)	1.09(0.91,1.31)	1.00	1.34(1.13,1.60)	1.18(0.99,1.41)	0.88
Gynecological cancers							
	Number of deaths	25	30	39	39	41	
	Age-adjusted hazard ratio	0.67(0.41,1.11)	0.77(0.48,1.24)	1.00	0.99(0.64,1.55)	1.02(0.66,1.58)	0.07
	Multivariate hazard ratio^b^	0.69(0.42,1.15)	0.78(0.48,1.25)	1.00	1.03(0.66,1.61)	1.07(0.68,1.67)	0.05
Digestive system cancers							
	Number of deaths	145	126	107	144	133	
	Age-adjusted hazard ratio	1.43(1.12,1.84)	1.19(0.92,1.54)	1.00	1.34(1.05,1.73)	1.17(0.91,1.52)	0.18
	Multivariate hazard ratio^b^	1.43(1.11,1.84)	1.18(0.91,1.53)	1.00	1.38(1.07,1.77)	1.21(0.94,1.57)	0.31
Respiratory system cancers							
	Number of deaths	64	51	45	59	52	
	Age-adjusted hazard ratio	1.51(1.03,2.22)	1.15(0.77,1.71)	1.00	1.32(0.89,1.94)	1.09(0.73,1.62)	0.13
	Multivariate hazard ratio^b^	1.49(1.01,2.18)	1.14(0.76,1.70)	1.00	1.33(0.90,1.96)	1.12(0.75,1.67)	0.20

Abbreviation: HR: hazard ratio; CIs: confidence intervals.

^*a*^Reference category.

^*b*^Adjusted for age at study enrollment, BMI, WHR, education, occupation, income, regular exercise (met/hour/year), current smoking (yes/no), current alcohol consumption (yes/no), marital status, age at menarche, and number of live births.

^*c*^ *P_trend_* for age at menopause (categorical).

In the multivariable models, younger age at menarche was associated with a trend of increased risk of mortality from all causes (Table 3). Compared with women aged 15 years at menarche, HRs and 95%CIs were 1.09 (0.97, 1.23) for women aged <14 years at menarche, 0.97 (0.86, 1.09) for women aged 14 years, 0.93 (0.83, 1.03) for women aged 16 years, and 0.94 (0.85,1.03) for women aged ≥17 years (*P*_trend_ = 0.01). Younger age at menarche was also associated with mortality from stroke. Compared with women aged 15 years at menarche, HRs and (95%CIs) were 1.23 (0.93,1.62) for women aged <14 years at menarche, 1.00 (0.75,1.33) for women aged 14 years, 0.99 (0.76,1.28) for women aged 16 years, and 0.87 (0.68,1.12 for women aged ≥17 years (*P*_trend_ = 0.03). Younger age at menarche was also associated with mortality from diabetes. Compared with women aged 15 years at menarche, HRs and (95%CIs) were 1.27 (0.81, 1.99) for women aged <14 years at menarche, 1.33 (0.88, 2.01) for women aged 14 years, 1.06 (0.72, 1.58) for women aged 16 years, and 0.84 (0.58, 1.23) for women aged ≥17 years (*P*_trend_ = 0.02). On the other hand, younger age at menarche was associated with a trend of lower risk of mortality from respiratory system cancers. Compared with women aged 15 years at menarche, HRs and (95%CIs) were 0.74 (0.48, 1.14) for women aged <14 years at menarche, 0.71 (0.47, 1.09) for women aged 14 years, 0.96 (0.67, 1.39) for women aged 16 years, and 1.15 (0.82, 1.61) for women aged ≥17 years (*P*_trend_ = 0.01).

**Table 3:** HRs (95%CIs) of age at menarche (years) with total and cause-specific mortality, Shanghai Women's Health Study.

Cause of death		Quintiles of age at menarche (years)					*P_trend_* c
		<14	14	15^a^	16	≥17	
		(*N* = 5,934)	(*N* = 5,784)	(*N* = 6,590)	(*N* = 6,178)	(*N* = 7,469)	
All causes							
	Number of deaths	503	492	646	629	888	
	Age-adjusted hazard ratio	0.99(0.88,1.12)	0.92(0.82,1.03)	1.00	0.96(0.86,1.07)	1.03(0.93,1.14)	0.32
	Multivariate hazard ratio^b^	1.09(0.97,1.23)	0.97(0.86,1.09)	1.00	0.93(0.83,1.03)	0.93(0.84,1.03)	0.01
All CVD							
	Number of deaths	150	150	198	216	287	
	Age-adjusted hazard ratio	1.01(0.81,1.25)	0.93(0.75,1.15)	1.00	1.05(0.87,1.28)	1.04(0.86,1.24)	0.42
	Multivariate hazard ratio^b^	1.14(0.92,1.41)	1.00(0.81,1.23)	1.00	1.02(0.84,1.24)	0.93(0.78,1.12)	0.11
Ischemic heart disease							
	Number of deaths	28	36	47	54	71	
	Age-adjusted hazard ratio	0.80(0.50,1.27)	0.95(0.61,1.46)	1.00	1.11(0.75,1.64)	1.07(0.74,1.56)	0.16
	Multivariate hazard ratio^b^	0.91(0.56,1.45)	1.01(0.65,1.56)	1.00	1.05(0.71,1.55)	0.93(0.64,1.35)	0.99
Stroke							
	Number of deaths	91	84	11	117	150	
	Age-adjusted hazard ratio	1.09(0.83,1.44)	0.93(0.70,1.24)	1.00	1.02(0.78,1.32)	0.96(0.75,1.23)	0.57
	Multivariate hazard ratio^b^	1.23(0.93,1.62)	1.00(0.75,1.33)	1.00	0.99(0.76,1.28)	0.87(0.68,1.12)	0.03
Diabetes							
	Number of deaths	33	45	46	55	67	
	Age-adjusted hazard ratio	0.98(0.62,1.53)	1.21(0.80,1.83)	1.00	1.15(0.77,1.69)	1.02(0.70,1.49)	0.97
	Multivariate hazard ratio^b^	1.27(0.81,1.99)	1.33(0.88,2.01)	1.00	1.06(0.72,1.58)	0.84(0.58,1.23)	0.02
All cancers							
	Number of deaths	215	210	293	254	349	
	Age-adjusted hazard ratio	0.90(0.75,1.07)	0.85(0.71,1.01)	1.00	0.87(0.74,1.03)	0.93(0.8,1.09)	0.65
	Multivariate hazard ratio^b^	0.94(0.78,1.12)	0.87(0.73,1.04)	1.00	0.85(0.72,1.01)	0.89(0.76,1.04)	0.48
Gynecological cancers							
	Number of deaths	30	29	37	33	45	
	Age-adjusted hazard ratio	0.94(0.58,1.52)	0.91(0.56,1.48)	1.00	0.92(0.58,1.48)	1.01(0.65,1.57)	0.74
	Multivariate hazard ratio^b^	0.90(0.55,1.46)	0.89(0.55,1.46)	1.00	0.91(0.57,1.46)	1.01(0.65,1.58)	0.63
Digestive system cancers							
	Number of deaths	109	112	150	123	161	
	Age-adjusted hazard ratio	0.90(0.70,1.15)	0.89(0.69,1.13)	1.00	0.82(0.65,1.04)	0.83(0.67,1.04)	0.37
	Multivariate hazard ratio^b^	0.95(0.74,1.23)	0.92(0.72,1.17)	1.00	0.80(0.63,1.02)	0.79(0.63,0.98)	0.06
Respiratory system cancers							
	Number of deaths	34	34	58	56	88	
	Age-adjusted hazard ratio	0.75(0.49,1.14)	0.71(0.46,1.08)	1.00	0.96(0.65,1.38)	1.15(0.82,1.68)	0.01
	Multivariate hazard ratio^b^	0.74(0.48,1.14)	0.71(0.47,1.09)	1.00	0.96(0.67,1.39)	1.15(0.82,1.61)	0.01

Abbreviation: HR: hazard ratio; CIs: confidence intervals.

^*a*^Reference category.

^*b*^Adjusted for age at study enrollment, BMI, WHR, education, occupation, income, regular exercise (met/hour/year), current smoking (yes/no), current alcohol consumption (yes/no), marital status, age at menopause, and number of live births.

^*c*^ *P_trend_* for age at menarche (categorical).

Women with a longer reproductive span had a significant trend of increased risk of mortality from gynecological cancers after adjustment for potential confounding factors (*P*_trend_ = 0.03, Table 4). Compared with women with a reproductive span of 30.16–32.45 years, HRs and 95%CIs were 0.66 (0.40, 1.11) for women with a reproductive span of <27.11 years, 0.84 (0.52, 1.35) for a span of 27.11–30.15 years; 1.24 (0.80, 1.94) for a span of 32.46–34.86 years, and 1.11 (0.68, 1.80) for a span of ≥34.87 years.

**Table 4:** HRs (95%CIs) of reproductive years with total and cause-specific mortality, Shanghai Women's Health Study.

Cause of death		Quintiles of reproductive years (years)					*P_trend_* c
		<27.11	27.11–30.15	30.16–32.45^a^	32.46–34.86	≥34.87	
		(*N* = 6,373)	(*N* = 6,404)	(*N* = 6,386)	(*N* = 6,394)	(*N* = 6,398)	
All causes							
	Number of deaths	730	703	625	582	518	
	Age-adjusted hazard ratio	1.11(0.99,1.25)	1.03(0.92,1.16)	1.00	1.01(0.90,1.14)	0.88(0.78,1.00)	<0.001
	Multivariate hazard ratio^b^	1.10(0.98,1.22)	1.03(0.92,1.15)	1.00	1.06(0.94,1.19)	0.99(0.88,1.12)	0.16
All CVD							
	Number of deaths	242	228	198	176	157	
	Age-adjusted hazard ratio	1.19(0.97,1.46)	1.04(0.85,1.28)	1.00	1.01(0.82,1.26)	0.86(0.69,1.09)	0.004
	Multivariate hazard ratio^b^	1.11(0.92,1.35)	1.03(0.85,1.24)	1.00	1.06(0.87,1.30)	1.03(0.83,1.28)	0.43
Ischemic heart disease							
	Number of deaths	50	58	51	45	32	
	Age-adjusted hazard ratio	0.91(0.60,1.39)	0.94(0.62,1.43)	1.00	1.03(0.67,1.59)	0.63(0.38,1.04)	0.41
	Multivariate hazard ratio^b^	0.87(0.58,1.30)	0.99(0.68,1.45)	1.00	1.09(0.73,1.64)	0.87(0.55,1.38)	0.64
Stroke							
	Number of deaths	126	121	109	98	98	
	Age-adjusted hazard ratio	1.14(0.86,1.49)	1.04(0.79,1.37)	1.00	1.02(0.76,1.37)	0.99(0.73,1.33)	0.30
	Multivariate hazard ratio^b^	1.05(0.81,1.37)	0.99(0.76,1.28)	1.00	1.07(0.81,1.41)	1.16(0.87,1.54)	0.61
Diabetes							
	Number of deaths	54	64	53	45	30	
	Age-adjusted hazard ratio	1.01(0.67,1.50)	1.05(0.71,1.56)	1.00	0.99(0.65,1.52)	0.62(0.38,1.01)	0.12
	Multivariate hazard ratio^b^	0.89(0.60,1.32)	1.05(0.73,1.52)	1.00	1.07(0.72,1.60)	0.85(0.54,1.35)	0.80
All cancers							
	Number of deaths	294	272	251	263	241	
	Age-adjusted hazard ratio	1.12(0.93,1.33)	0.99(0.83,1.19)	1.00	1.07(0.89,1.28)	0.99(0.82,1.19)	0.27
	Multivariate hazard ratio^b^	1.13(0.95,1.34)	1.02(0.86,1.22)	1.00	1.13(0.95,1.35)	1.04(0.87,1.26)	0.52
Gynecological cancers							
	Number of deaths	25	32	37	43	37	
	Age-adjusted hazard ratio	0.60(0.36,1.00)	0.79(0.49,1.27)	1.00	1.08(0.69,1.68)	0.93(0.59,1.48)	0.04
	Multivariate hazard ratio^b^	0.66(0.40,1.11)	0.84(0.52,1.35)	1.00	1.24(0.80,1.94)	1.11(0.68,1.80)	0.03
Digestive system cancers							
	Number of deaths	153	131	126	134	111	
	Age-adjusted hazard ratio	1.18(0.92,1.53)	1.02(0.78,1.32)	1.00	1.12(0.86,1.46)	0.94(0.71,1.24)	0.16
	Multivariate hazard ratio^b^	1.21(0.95,1.53)	1.00(0.78,1.28)	1.00	1.13(0.89,1.45)	0.93(0.71,1.21)	0.11
Respiratory system cancers							
	Number of deaths	65	72	47	40	47	
	Age-adjusted hazard ratio	1.31(0.90,1.91)	1.42(0.98,2.05)	1.00	0.93(0.61,1.42)	1.07(0.71,1.60)	0.07
	Multivariate hazard ratio^b^	1.25(0.85,1.83)	1.36(0.94,1.98)	1.00	0.97(0.63,1.48)	1.15(0.75,1.76)	0.35

Abbreviation: HR: hazard ratio; CIs: confidence intervals.

^*a*^Reference category.

^*b*^Adjusted for age at study enrollment, BMI, WHR, education, occupation, income, regular exercise (met/hour/year), current smoking (yes/no), current alcohol consumption (yes/no), marital status, age at menarche, and number of live births.

^*c*^ *P_trend_* for number of reproductive years (categorical).

Older age at menarche was associated with higher risk of mortality from lung cancer (*P*_trend_ = 0.02), and a similar association was observed among non-smokers (*P*_trend_ = 0.02). However, no other statistically significant associations were found in the current study between the three menstrual history variables and mortality from breast, endometrial, colorectal, or gastric cancers (data not shown).

## Discussion

Results from this large, prospective cohort study of Chinese women aged 40–70 years suggest that younger age at menopause was associated with higher total mortality. Early menarche was associated with higher risk of mortality from all causes, stroke, and diabetes, but with lower risk of mortality from respiratory system cancers. A longer reproductive span was associated with higher mortality from gynecological cancers.

Our result that early menopause increases total mortality is consistent with results from most previous studies on this topic [2], [4], [5], [17], [21], which supports the hypothesis that early natural menopause is a general indicator of premature aging [22]. In line with results from studies in Western populations [9], [11], [23], an inverse association between age at menarche and total mortality was also found in our study. Our findings that both late menarche and late natural menopause were associated with lower risk of total mortality adds further support to previous findings that women who are biologically younger than their chronologic age (have late menarche or late natural menopause) have lower mortality than women with an average age at menarche or natural menopause [23].

In the Seventh-Day Adventist study in California, postmenopausal women were categorized into groups according to age at menarche (<12, 12, 13, 14, 15, >15). Each increase in category of age at menarche was observed to decrease risk of stroke mortality by 9.6% (95%CI:0.4–18.6) after adjustment for BMI, physical activity, age at first birth, type of menopause, and use of HRT [11]. Similar to this finding, late menarche was associated with lower risk of stroke and diabetes mortality in our study. Many studies have shown that early menarche is associated with increased cardiovascular disease, possibly due to the increased body fatness in childhood [24]. Early menarche has also been associated with increased risk of diabetes in adults [25], one of the main risk factors for stroke [26]. Consequently, the inverse association between age at menarche and stroke mortality could also partly be explained by the inverse association between age at menarche and diabetes risk.

Late menarche was related to high risk of mortality from respiratory system cancers in our study. Our results also indicated that lung cancer mortality contributed to this association. Studies examining late menarche and lung cancer risk have been inconsistent, indicating either no significant effect or decreased risk [27]–[30]. To our knowledge, the association of late menarche with higher lung cancer mortality is a novel finding and needs to be confirmed in further studies.

Among 68,154 US women who experienced natural menopause and had not used tobacco or HRT, Mondul *et al.* observed that mortality from breast or ovarian cancer was lower among women whose age at menopause was 40–44 years (rate ratio [95%CIs]: 0.68 [0.56, 0.82] or 45–49 years (0.93 [0.83, 1.03]), compared with women whose age at menopause was 50–54 years [3]. Although no significant association between age at menopause and gynecological cancer mortality was found in our study, we observed that having a shorter reproductive span was associated with lower risk mortality from gynecological cancers. It has been suggested that the inverse association between age at menopause and gynecological cancer risk is due to the cessation of cyclical ovarian hormone production at menopause [31]–[33]. The shorter the reproductive span, the lower the cumulative lifetime exposure to endogenous hormones would be in these women. Consequently, the reduction in circulating hormone concentrations might lead to a reduction in relative risk for gynecological cancers. The ‘U-shaped’ relationship between age at menopause and mortality from all cancers observed in the current study appears to be related to death due to an excess of digestive and respiratory system cancers among women with early age at menopause and an excess of gynecological cancers for women with late age at menopause. More studies are warranted to investigate the role of sex hormone in the etiology and prognosis of these cancers.

Strengths of this study include: the prospective design, the large population-based sample size, and the high response rates. In addition, the current study was restricted to women who had natural menopause and had never used HRT. The main limitation of this study is that ages at menarche and menopause were self-reported and may, therefore, be subject to recall bias. However, previous studies have reported that the recall of age at menarche and menopause is relatively accurate [34], [35]. In addition, although *P*-values for the trend tests were significant, many point estimates were not statistically significant and confidence intervals included 1.00. It should also be noted that our study involved multiple comparisons which may have led an increase of false positive findings (inflated type 1 error). Moreover, event numbers for some of the sub-group analyses were low, and thus, some results could have been due to chance findings. Furthermore, due to the differences in dietary and environmental exposures, the findings of our study may not be directly generalizable to other populations.

In summary, this study of Chinese women found inverse associations for age at menarche and natural menopause with total mortality and for age at menarche with mortality from stroke and diabetes. However, we found positive associations for age at menarche with mortality from respiratory system cancers, as well as for duration of the reproductive span with mortality from gynecological cancers. Our results demonstrate that women who experience early menarche or early natural menopause tend to have increased total mortality.
